# Elucidating the differences in oxidation of high-performance α- and β- diisobutylene biofuels via Synchrotron photoionization mass spectrometry

**DOI:** 10.1038/s41598-020-76462-y

**Published:** 2020-12-11

**Authors:** Anthony Carmine Terracciano, Sneha Neupane, Denisia M. Popolan-Vaida, Richard G. Blair, Nils Hansen, Ghanshyam L. Vaghjiani, Subith S. Vasu

**Affiliations:** 1 Mechanical and Aerospace Engineering Department, University of Central Florida, 4000 Central Florida Blvd., Orlando, FL 32816-2450 USA; 2 Center for Advanced Turbomachinery and Energy Research (CATER), University of Central Florida, 4000 Central Florida Blvd., Orlando, FL 32816-2450 USA; 3 Department of Chemistry, University of Central Florida, 4000 Central Florida Blvd., Orlando, FL 32816-2450 USA; 4 Florida Space Institute, University of Central Florida, 4000 Central Florida Blvd, Orlando, FL 32816-2450 USA; 5 Combustion Research Facility, Sandia National Laboratories, MS 9055, P.O. Box 969, Livermore, CA 94551 USA; 6 In-Space Propulsion Branch, Rocket Propulsion Division, Aerospace Systems Directorate, Air Force Research Laboratory, AFRL/RQRS, Edwards AFB, CA 93524 USA; 7 Present Address: Oak Ridge National Lab, Oak Ridge, TN USA

**Keywords:** Mechanical engineering, Chemical engineering

## Abstract

Biofuels are a promising ecologically viable and renewable alternative to petroleum fuels, with the potential to reduce net greenhouse gas emissions. However, biomass sourced fuels are often produced as blends of hydrocarbons and their oxygenates. Such blending complicates the implementation of these fuels in combustion applications. Variations in a biofuel’s composition will dictate combustion properties such as auto ignition temperature, reaction delay time, and reaction pathways. A handful of novel drop-in replacement biofuels for conventional transportation fuels have recently been down selected from a list of over 10,000 potential candidates as part of the U.S. Department of Energy’s (DOE) Co-Optimization of Fuels and Engines (Co-Optima) initiative. Diisobutylene (DIB) is one such high-performing hydrocarbon which can readily be produced from the dehydration and dimerization of isobutanol, produced from the fermentation of biomass-derived sugars. The two most common isomers realized, from this process, are 2,4,4-trimethyl-1-pentene (α-DIB) and 2,4,4-trimethyl-2-pentene (β-DIB). Due to a difference in olefinic bond location, the α- and β- isomer exhibit dramatically different ignition temperatures at constant pressure and equivalence ratio. This may be attributed to different fragmentation pathways enabled by allylic versus vinylic carbons. For optimal implementation of these biofuel candidates, explicit identification of the intermediates formed during the combustion of each of the isomers is needed. To investigate the combustion pathways of these molecules, tunable vacuum ultraviolet (VUV) light (in the range 8.1–11.0 eV) available at the Lawrence Berkeley National Laboratory’s Advanced Light Source (ALS) has been used in conjunction with a jet stirred reactor (JSR) and time-of-flight mass spectrometry to probe intermediates formed. Relative intensity curves for intermediate mass fragments produced during this process were obtained. Several important unique intermediates were identified at the lowest observable combustion temperature with static pressure of 93,325 Pa and for 1.5 s residence time. As this relatively short residence time is just after ignition, this study is targeted at the fuels’ ignition events. Ignition characteristics for both isomers were found to be strongly dependent on the kinetics of C_4_ and C_7_ fragment production and decomposition, with the tert-butyl radical as a key intermediate species. However, the ignition of α-DIB exhibited larger concentrations of C_4_ compounds over C_7_, while the reverse was true for β-DIB. These identified species will allow for enhanced engineering modeling of fuel blending and engine design.

## Introduction

Sustainability and net carbon emissions for transportation fuel, is of critical importance as anthropogenic emissions continue to rise. Today, the most common method of powering transportation is through combustion of nonrenewable fossil fuels. Biofuels produced from processed waste and non-food feedstocks may be implemented either neat or blended components into existing fuels to reduce reliance on liquid fossils with little change to the existing infrastructure^1,2^. Through choice, biofuels can exhibit similarities in their composition and other physical and chemical properties to conventional fossil fuels while potentially reducing net carbon emissions. One example of a biofuel that has been successfully blended with fossil fuels is ethanol and as a result, the focus of biofuel combustion research during the last 3 decades has mainly been on ethanol blends^3–15^.

**Figure 1:**
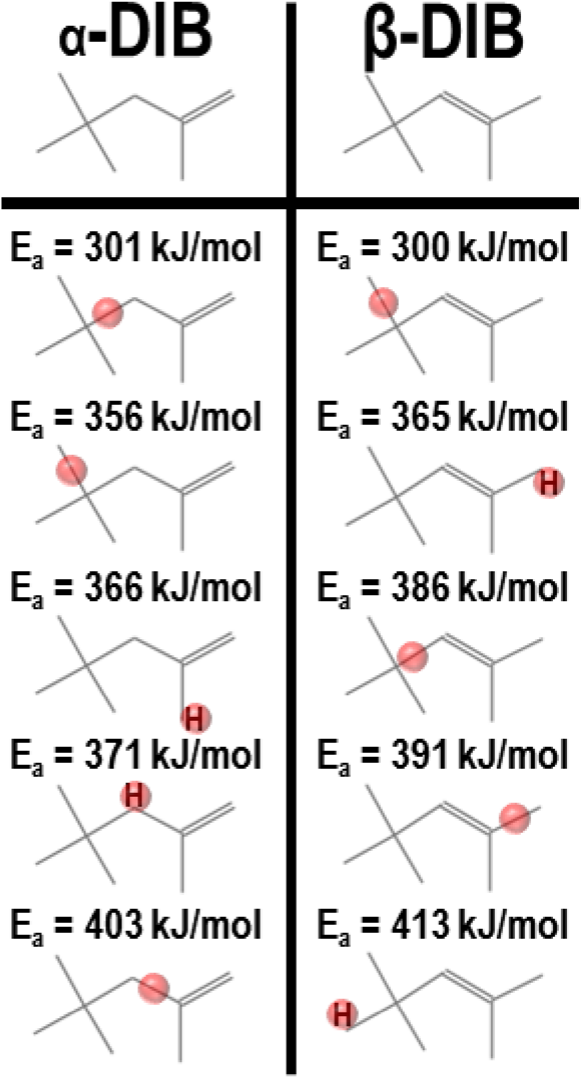
Diisobutylene α-, β- shown with the placement of C=C bond^27^ and five weakest bond dissociation energies^29^.

However, while ethanol is advantageous, producing little soot^16^, it has several disadvantages among which are its relatively low energy density, high combustion temperature, poor seal compatibility, and hydrophilicity. Over the last decade (2010–2020), researchers have examined a variety of other biofuels to replace or augment fossil fuels. To this end, the U.S. Federal Government has initiated the Co-Optima Project, which seeks to explore and implement different fuel feedstocks for transportation based internal combustion engines^17^. Co-Optima aims to utilize existing infrastructure systems to complement novel energy conversion methods for future vehicles to obtain carbon neutral transportation^18–20^. One fuel of interest under Co-Optima is diisobutylene (DIB), which can be produced from the dehydration and dimerization of isobutanol realized from the fermentation of biomass-derived sugars by *Saccharomyces cerevisiae*^21^. The dehydration and isomerization process is performed over acidic catalysts, which produces two isomers: 2,4,4-trimethyl-1-pentene (α-DIB) and 2,4,4-trimethyl-2-pentene (β-DIB) (Fig. 1)^22,23^. DIB has been shown to have several properties which are advantageous for use as a transportation fuel such as: (i) dramatic reductions in NO output over iso-octane^24^, (ii) stability to oligomerization and polymerization even when exposed to sunlight^25^, and (iii) compatibility with existing seal and O-ring materials^26^. However, common production methods of DIB result in a blend of 75 vol% α-DIB (Fig. 1, left) and 25 vol% β-DIB (Fig. 1, right)^27^. With a formation enthalpy difference of only 3.51 kJ/mol, the production of a single isomer during this process is not currently cost effective^28^.

From Fig. 1, it can be seen that the location of the olefinic carbons differentiates the two isomers. Aside from the shown variations in the values for the bond dissociation energy (BDE) for these molecules^29^, their structural changes are observable through non-reactive methods including infrared (IR) absorption spectroscopy^30^. Such changes in bond placement has been shown to drastically modify the ignition characteristics in other molecules, and DIB is no exception^31^. Shock tube ignition studies have shown a drastically lowered ignition delay for neat β-diisobutylene^32^, and it is well known that the β-isomer will ignite at a significantly lower temperature than does the α-isomer with all else being equal.

However, there have been no studies that have investigated the reaction kinetics of neat DIBs during ignition using a technique that can probe the individual DIB isomers during their low temperature oxidation. A highly selective and sensitive means for determining, in situ, the reaction kinetics in such systems is by the application of photoionization mass spectrometry^33^. In this work, a jet stirred reactor (JSR) is used in conjunction with Synchrotron radiation from the Advanced Light Source (ALS) at the Lawrence-Berkeley National Laboratory to carry out mass spectrometric measurements. Stoichiometric mixtures of diisobutylene isomers and oxygen (O_2_) with Ar as a diluent at a fixed reactor pressure (P_0_) of 93,325 ± 333 Pa are sampled at time τ = 1500 ± 50 ms following injection at the lowest observed temperature for which ignition could be observed. The mass spectrometer used in this work had a mass resolution of m/Δm = 2500^34^, while the Synchrotron light had a bandwidth of ± 0.05 eV. The technique of multiplexed photoionization mass spectrometry was utilized to detect the individual isomers and reaction intermediates by analyzing m/z spectra between 40 and 130 amu.

## Methods: JSR and the Synchrotron beam

Introduction of reactants and diluents comprised of pressure regulated high purity (> 99.999%) O_2_ and Ar gaseous flows into the JSR, which was externally heated via an oven and held at a fixed temperature. Gases were metered using calibrated mass flow controllers from MKS Instruments (Andover, USA). O_2_ was metered using a controller with 500 sccm maximum flow. Ar was metered using both a 100 and a 1000 sccm maximum flow controllers, which respectively, diluted the fuel and oxidizer streams. Liquid DIB fuel stream comprised of either 99% 2,4,4,-trimethyl-1-pentene (α-DIB) or 99% 2,4,4,-trimethyl-2-pentene (β-DIB) (Sigma-Aldrich, St. Louis, USA), was injected via a hypodermic syringe through a septum that enabled the fuel to be carried towards the JSR by the externally heated Ar flow at 380 K. Such a dilution scheme by excess Ar of both the O_2_ and the liquid fuel streams provided control of the duration of the combustion reaction (residence) time, τ before being sampled.

**Figure 2:**
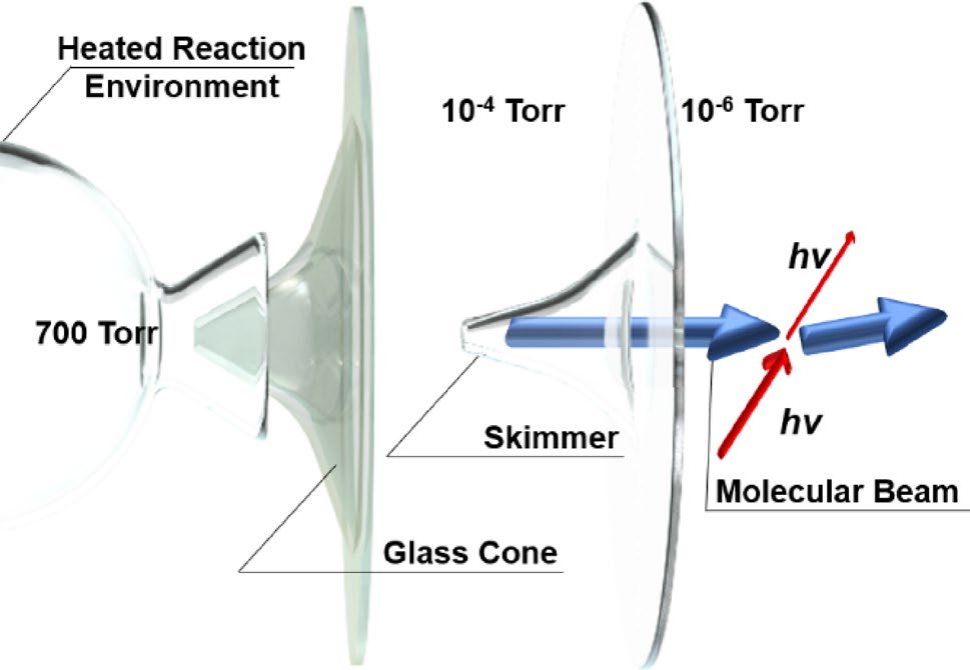
Configuration of the jet stirred reactor (JSR) and the mass spectrometer expansion stages for use with the Synchrotron beam (hν).

A set of four specifically oriented and positioned injection nozzles then delivered the reactants into the isobaric and isothermal JSR. Upon the discharge of the reacted mixture from the JSR, a glass sampling cone was used to continuously draw a small portion of the mixture from the JSR source into the 1st stage expansion volume of the mass spectrometer chamber held at 10^–4^ Torr. Such a low pressure ensured a long mean free path between the molecules and/or radicals sampled thereby “freezing” their composition. From this 10^–4^ Torr chamber, a skimmer then sampled the mixture into the 2nd stage expansion volume held at 10^–6^ Torr to form a molecular beam of the reacted mixture that was made to intersect the tunable VUV radiation source (see Fig. 2). Photon absorption resulted in the ionization of molecules and radicals with some characteristic intensity. The ionized species were separated in a time of flight tube by their m/z values and counted using a microchannel plate (MCP) detector^33^. For this work, a photon energy range from 8.10 to 11.00 eV (bandwidth ± 0.05 eV), with an incremental step taken at the bandwidth limit, was used with data from 2^19^ mass spectrometer recordings being co-added at each of the energy incremental to collect the mass spectra. Further details of the JSR method can be found elsewhere^34,35^. An extensive review of the Synchrotron source and the collection of the photoionization data can also be found elsewhere^33,36–42^.

Oxidation conditions, including reaction temperatures, for the two DIB isomers are presented in Table 1. Data corresponding to the resolved mass channels from the JSR-ALS were integrated to enable subsequent photoionization spectral investigations. This data was then complemented with available photoionization cross sections (PICS) from existing literature and databases^43–79^. While a significant amount of qualitative information can be extracted from the present experimental data, the absolute mole fraction representation was not possible due to the absence of some PICS literature values^80^.

**Table 1:** Flow configuration for experiments using α- and β-diisobutylene (DIB).

Fuel	T [K]	τ [ms]	P_s_ [Torr]	Ar-fuel [sccm]	Ar-O_2_ [sccm]	O_2_ [sccm]	Fuel [μl/min]	Φ
α-DIB	700	1500	700	100.0	324.6	58.6	34	1.00
β-DIB	620	1500	700	100.0	379.3	66.1	38	1.00

## Results and discussion

Results from the experiments conducted herein include relative intensity curves for both α- and β-DIB. Intensity refers to the ratio of the observed signal at any given mass and the photon energy compared to that of the maximum observed signal at 10.5 eV for each of the experiments^81^. In both experiments, the scalar value used is from the 10.5 eV exposure of the mass channel assigned to DIB.

### Photoionization intensity curves of α- & β-diisobutylene (DIB)

**Figure 3:**
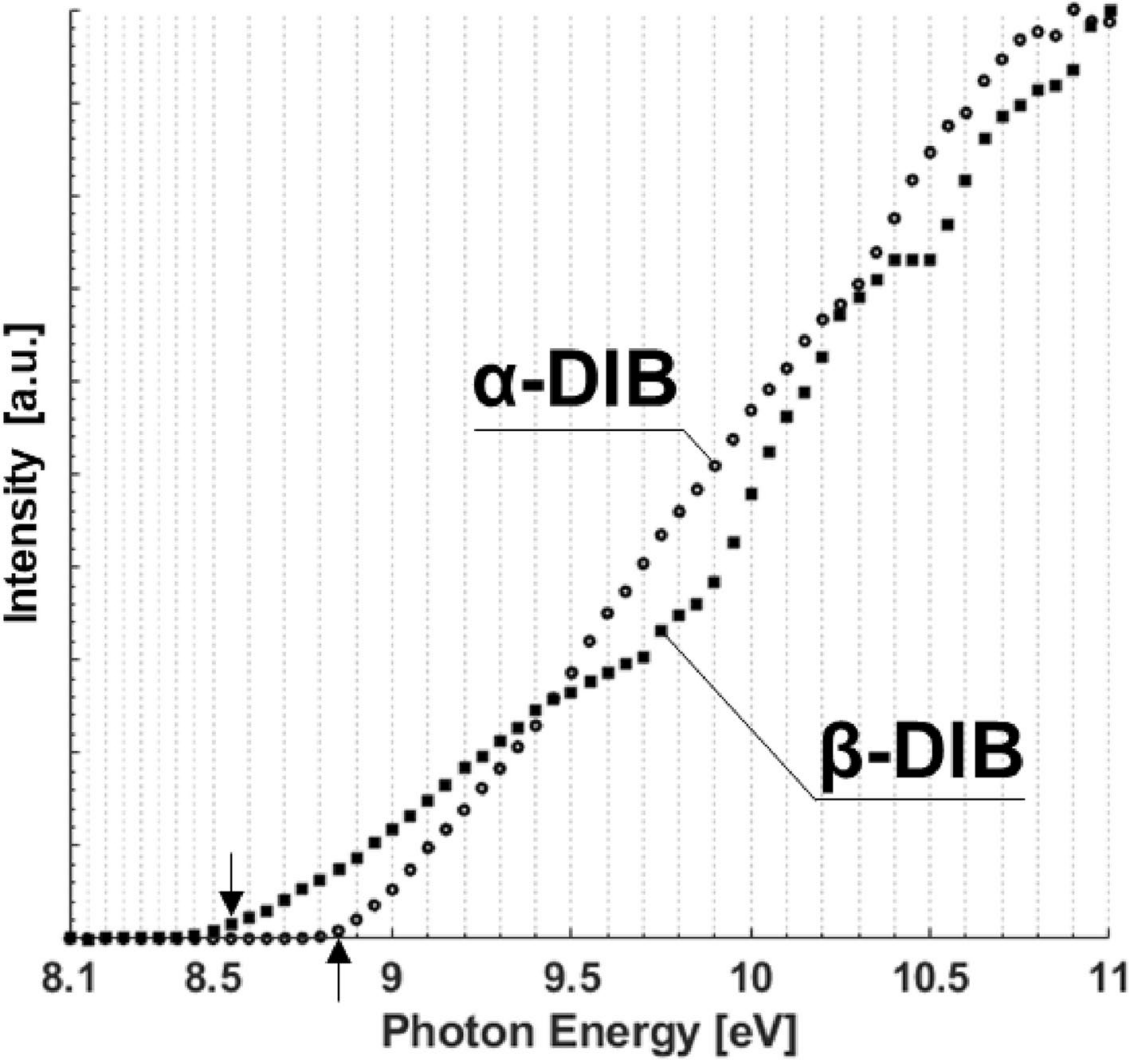
Relative intensity of α- & β-DIB. Arrows indicate the corresponding IE of each at 112.21 amu.

With photoionization mass spectrometry, it is possible to identify the presence of a given molecule accounting for both its isotopologue and isotopomer variations. Within Fig. 3, for both the α- and β-DIB isomer, the intensity curves for the most common isotopomer consisting of ^12^C_8_^1^H_16_, may be seen. The intensity curves for each isomer of DIB (α- or β-) are presented for both isotopologues, C_8_H_16_ and ^12^C_7_^13^C^1^H_16_, in the Supporting Info. Due to the low natural abundance of deuterium (D), isotopologues of α- and β-DIB containing D are neglected.

From Fig. 3 it can be seen that there are unique features in the intensity curves of the DIB isomers. β-DIB exhibits a significantly lower ionization energy (IE) of 8.55 eV *vs.* that of α-DIB’s IE of 8.85 eV. Examining the intensity, the α-DIB exhibits a near-linear monotonic rise from its IE until 10.20 eV. However, at 10.35 eV there is an apparent jump in the intensity and increases until 10.50 eV. Beyond 10.5 eV, the α-DIB intensity levels off until 11.00 eV. The intensity curve of β-DIB is significantly more complex featuring a variable monotonic rise between its IE and 9.35 eV, beyond which a reduced rise rate occurs until 9.90 eV, before a pronounced jump and a leveling trend of the intensity until 10.50 eV, beyond which a second sharper jump can be seen continuing until 10.90 eV.

### Photoionization mass spectra of α- & β-DIB ignition

**Figure 4:**
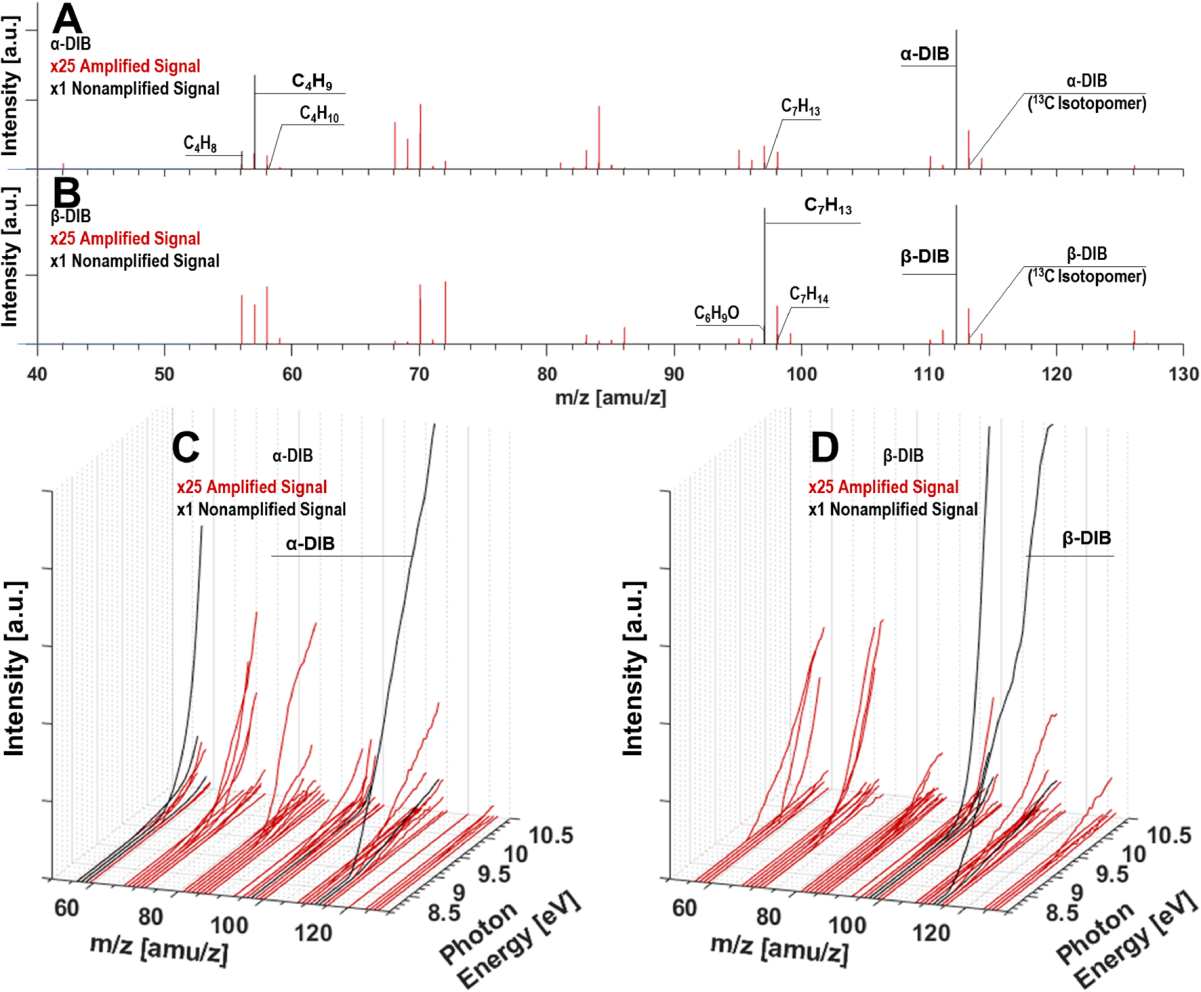
Intensity plots for α- and β-DIB: (**A**) and (**B**) at 10.5 eV photoionization, and (**C**) and (**D**) shown isometrically as a function of photon energy. Signals in red are multiplied by a factor of 25. *See this figure in the supporting info for enhanced clarity.

The photoionization mass spectra of the DIB combustion reaction mixture for conditions described in Table 1 for both the α- and β-isomer can be seen in Fig. 4. Figure 4A,B display m/z data for ionization with 10.50 eV VUV light, while Fig. 4C,D display the intensity along each m/z channel (x-axis) and photon energy (y-axis) simultaneously. For the data presented in Fig. 4 as well as for all subsequent Tables and Figures, the intensity has been normalized with respect to the mass channel m/z = 112.2 corresponding to the parent DIB isomer ion signal^81^. Observation of this ion, in such quantity, indicates success in probing the reaction shortly after ignition. In Fig. 4A,B these are denoted as black bars labeled “ × 1 Nonamplified Signal.” To further enhance the visibility of the minor m/z channels with low relative intensity values (from 4 to 40 × 10^–3^), their data is denoted as red bars that have been amplified by a factor of 25 in the figure and labeled “ × 25 Amplified Signal.” Signals below the threshold of 40 × 10^–3^ were omitted for consideration in the present study. We note that to adequately investigate m/z channels that have intensity values below 40 × 10^–3^, a scan at each energy discretization with sampling much more than 2^19^ co-additions would be required; hence their neglection from this study. While the CO_2_ signal (m/z = 43.99) is expected to be within the sampled mass range, its IE is in excess of 13 eV and therefore is not detectable with the range of VUV light used in this work^82^.

In Fig. 4A,B, there are several m/z signals that are common to the combustion of both isomers. Common signal groupings can be identified for m/z ranges of 56–60, 68–72, 81–87, 95–100, 110–105, as well as one possible grouping of 120–127 for each of the DIB isomers. As this latter window is above the fuel’s molecular weight by 8–15 amu, it is highly likely that these species are the result of either: (i) reactions of pairs of species containing C_3-5_; or (ii) reactions by smaller radicals including CHO, OH, H, etc., with species containing C_6-7_; or (iii) reactions of the fuel molecule with oxygen. A more detailed view of these spectra is presented within the Appendix (see Supporting Info) with each of the m/z channels labeled with corresponding isomer compositions.

The combined presentation of information offered through Fig. 4A,C, and Fig. 4B,D give a more comprehensive view of the sampled intermediate species resulting from fuel oxidation reactions. For the α-DIB reaction (see Fig. 4A,C), there are four m/z compositions that are not considered “minor” relative to α-DIB and its common isotopologue (C_7_^13^CH_16_): C_7_H_13_, C_4_H_10_, C_4_H_9_ and C_4_H_8_. For the β-DIB reaction, there are three m/z channels relative to the parent and its isotopologue (C_7_^13^CH_16_) that are considered major: C_7_H_14_, C_7_H_13_, and C_6_H_9_O. Large intensity values for these m/z indicate that these species are likely important in the ignition of their respective DIB isomers.

It is of importance to note that C_7_H_13_ being a primary intermediate for both isomer reactions may be formed via a methyl group cleaved from the parent DIB. The higher C_7_H_13_ intensity in β-DIB may be an indication of the increased role played by the additional methyl group at the C=C bond, though contribution from C_6_H_9_O, a likely by-product of DIB reactions cannot be ruled out. The C_4_H_9_ signal is identified as due to the butyl radical. Thus, any future mechanisms for these fuels should be validated against the kinetic traces for these species as well as C_4_H_8_ formed in perfectly stirred reactors.

### Identified intermediates of DIB ignition

In accordance with a previously reported method^35^, it is possible to use the PICS of known species to obtain a ratio of the target ion signal versus the reference ion signal as shown in Eq. (1), where the subscript-*i* denotes the sample species and subscript-*R* denotes the reference species. In Eq. (1), the signal, $S$, is a function of the species partial pressure, *P*, the wavelength-specific photoionization cross section $\upsigma (\mathrm{E})$ and the mass dependent response factor, $R,$ which depends on the instrumentation in use. In this work, it is assumed that values of $R$ are unity.

1 $$ \frac{{S_{i} }}{{S_{R} }} = \left[ {\frac{{P_{i} }}{{P_{R} }}} \right]\cdot\left[ {\frac{{\sigma_{i} \left( E \right)}}{{\sigma_{R} \left( E \right)}}} \right]\cdot\left[ {\frac{{R_{i} }}{{R_{R} }}} \right] $$

An abstraction of Eq. (1) is implemented that allows the use of relative intensity. This is done by using PICS for each isomer, taken from the literature σ (E) (see references within Tables 2 and 3 for each species), and is shown in Eq. (2). PICS for the given species is multiplied by its Scaling factor $SF$, to give the synthetic signal *SS*_*i*_*(E)* for the target species. These individual values of *SS*_*i*_(E) are added as in Eq. (3) to give a synthetic signal *SS*_*m/z*_*(E)* for the given mass channel which is then compared to the observed intensity at each m/z and eV combination. For each $SF$ value, an error of ± 30% is assigned^35,83^. This analysis was done for 5 non-oxygenated intermediate compositions and 4 oxygenated intermediate compositions.

**Table 2:** Oxygenated intermediates identified in DIB reactions with respective scaling factors and gas-phase heat of formation values.

Composition	ID #	Isomer name	$SF$[a.u. · 10^3^]		ΔH_f-(g)_ [kJ/mol]	Ref
			α-DIB	β-DIB		
C_3_H_6_O	*1*	2-Propen-1-ol	11.8	47.7	− 171.8	^43,44^
	*2*	Acetone	13.1	67.8	− 216.5	^45,46^
	*3*	Propanal	5.1	27.0	− 188.7	^47,48^
	*4*	Propen-2-ol	0.1	29.0	− 176.0	^49,84^
	*5*	Methyloxirane	11.0	69.7	− 94.7	^52,85^
C_4_H_6_O	*6*	2,3-Dihydrofuran	8.4E−3	55.7E−3	− 72.3	^43,53^
	*7*	2,5-Dihydrofuran	2.1	2.7	− 61	^43,86^
	*8*	2-Butenal	24.1	63.8	− 109.7	^43,74^
	*9*	Dimethylketene	33.7E−3	45.6	− 91.0	^75,76^
	*10*	Methyl Vinyl Ketone	5.3	11.2	− 115.0	^43,79^
	*11*	Ethylketene	0.3E−3	0.4E−3	− 86.5	^75,76^
	*12*	Methacrolein	19.4	26.8	− 106.4	^74,75^
C_4_H_8_O	*13*	2-Buten-1-ol	5.0	105.8	− 160.9	^50,77^
	*14*	3-Buten-1-ol	3.1	2.4	− 152.7	^50,77^
	*15*	Isobutanal	1.0	4.7	− 215.8	^43,48^
	*16*	Tetrahydrofuran	5.1	2.4	− 182.4	^47,61^
	*17*	n-Butanal	6.0	27.6	− 211.8	^43,48^
C_5_H_8_O	*18*	3-Penten-2-one	9.9	1.5	− 136.0	^56,87^
	*19*	Cyclopentanone	0.1	0.3	− 197.4	^48,60^
	*20*	3-Methyl-2-Butenal	4.8	0.3	− 124.7	^43,50^
C_5_H_10_O	*21*	2-Methyltetrahydrofuran	2.2	3.8	− 218.1	^43,50^
	*22*	3-Methylbutanal	12.5	60.2	− 236.5	^77,88,89^
	*23*	Isoprenol	29.3E−3	4.9	− 183.1	^50,78^
	*24*	Prenol	29.3E−	1.6	− 191.3	^50,77^
	*25*	Tetrahydropyran	0.5	2.2	− 223.8	^43,61^

**Table 3:** Non-oxygenated intermediates identified in DIB reactions, respective scaling factors, and gas-phase heat of formation values.

Composition	ID #	Isomer name	$SF$[a.u. · 10^3^]		ΔH_f-(g)_ [kJ/mol]	Ref
			α-DIB	β-DIB		
C_3_H_6_	*A*	Cyclopropane	14.2	8.1	39.3	^58,62^
	*B*	Propene	9.9	1.3	20.4	^57,58^
C_4_H_8_	*C*	1-Butene	136.4	21.6	− 0.6	^47,63^
	*D*	Cis-2-butene	31.0	10.8	− 7.7	^47,63^
	*E*	Isobutene	62.0	21.6	− 17.9	^47,63^
	*F*	Trans-2-butene	31.0	10.8	− 10.8	^43,63^
C_5_H_10_	*G*	1,1-Dimethyl-Cyclopropane	24.7	10.9	− 8.2	^64,65^
	*H*	1-Pentene	7.6	0.3	− 22.9	^47,66^
	*I*	2-methyl-1-butene	2.5	3.3	− 35.1	^43,48^
	*J*	2-methyl-2-butene	1.0	3.3	− 41.5	^43,48^
	*K*	3-methyl-1-butene	1.0	11.2	− 25.5	^43,67^
	*L*	Cyclopentane	3.7	4.9	− 76.4	^45,68^
	*M*	Ethyl-Cyclopropane	122.0	23.0	− 51.0	^64,90,91^
	*N*	Cis-2-pentene	0.2	1.7	− 28.0	^66,69^
	*O*	Trans-2-pentene	0.2	1.7	− 32.0	^66,69^
C_6_H_12_	*P*	1-Hexene	27.0	1.9	− 42.0	^43,66^
	*Q*	2,3-Dimethyl-2-Butene	0.2	93.4	− 70.3	^62,70^
	*R*	3,3-Dimethyl-1-Butene	77.3	0.7	− 60.5	^47,92^
	*S*	Cyclohexane	29.8	2.6	− 123.1	^62,71^
	*T*	Methylcyclopentane	3.6	3.0	− 106.0	^47,72^
	*U*	Trans-2-hexene	0.5	1.4	− 51.0	^43,73^

2 $${SS}_{i}(E)={SF}_{i}\cdot {\sigma }_{i}(E)$$

3 $${SS}_{m/z}(E)=\sum S{S}_{i}(E)$$

#### Oxygenated intermediates

**Figure 5:**
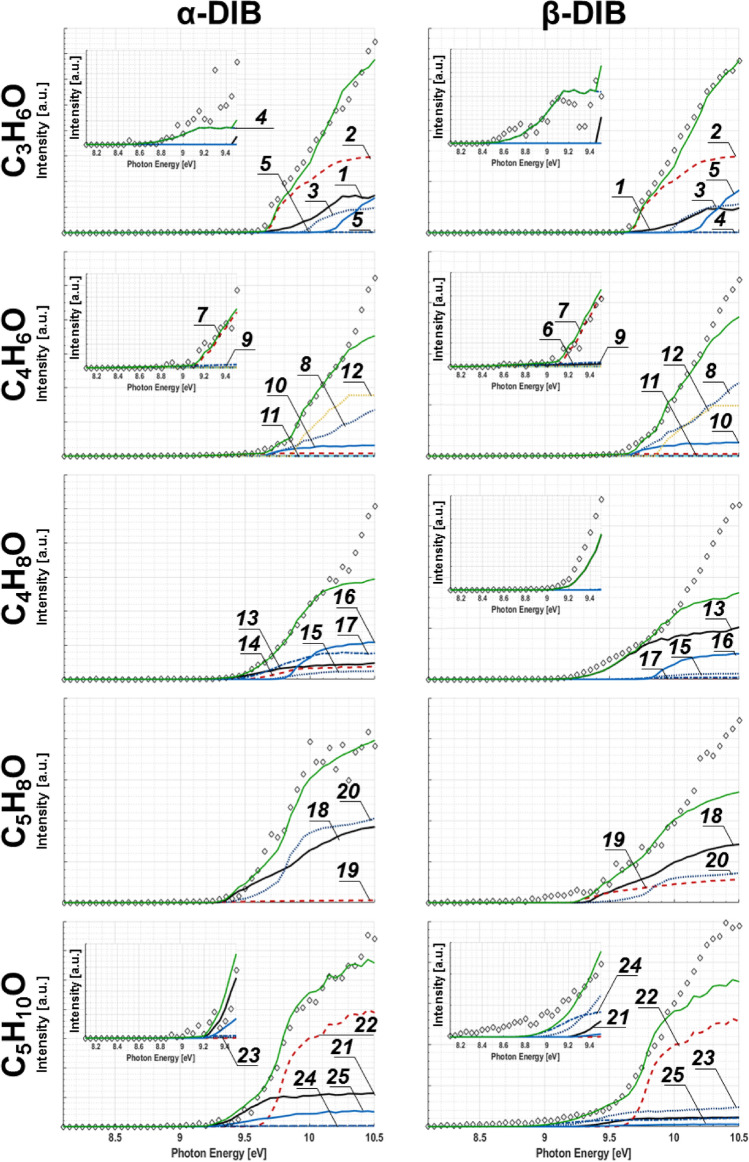
Intensity curves measured “◇” for oxygenated species in α- and β-DIB reactions. Colored traces correspond to those for species from Table 2, while green is reserved for the fit to experiment. Where appropriate, the inserts show magnified plots.

Both isomers of DIB produce discernable intensities corresponding to: C_3_H_6_O, C_4_H_6_O, C_4_H_8_O, C_5_H_8_O and C_5_H_10_O. From these mass channels, 25 individual isomers have been considered as being present within the respective reactions as sampled, and are shown in Table 2 along with the corresponding $SF$ values and values for the standard enthalpy of formation in the gas phase (ΔH_f-(g)_). As each of the corresponding isomers have gained an oxygen from a non-oxygenated fuel species, it may be concluded that these are byproducts in reactions that introduce an O. In Fig. 5, the experimentally measured intensity, *SS*_*i*_(E) (colored plots), and *SS*_*m/z*_*(E)* (green curves) for each species identified in Table 2 can be seen.

Each oxygenated species corresponds to a “minor” m/z channel. Additionally, in some of the plots in Fig. 5, it can be seen that at higher eV, there is a deviation between the *SS*_*m/z*_*(E)* and measured intensity. The reasons being: (i) some of the reference PICS of isomers do not have measured σ (E) values at energies above 10.5 eV, and (ii) it is possible that there are unaccounted isomers at this mass channel. An example of the limitation by (i) above can be seen in Fig. 5 for C_4_H_6_O, wherein the reference PICS of methacrolein cuts off at 10.30 eV, and the value observed at 10.30 eV is repeated, remaining constant until 10.50 eV. Unaccounted isomers are likely present in the C_4_H_8_O and C_5_H_10_O plots of Fig. 5, as there is a significant mismatch between the intensity and *SS*_*m/z*_* (E)* beyond 10.20 eV for the C_4_H_8_O trace and beyond 9.70 eV for the C_5_H_10_O trace.

For C_5_H_10_O and C_5_H_8_O plots, the most populated isomers identified are 3-methylbutanal and 3-penten-2-one, respectively, for both the α- and β-DIB reactions. When analyzing *SS*_*m/z*_*(E)* corresponding to C_5_H_10_O and C_5_H_8_O with that of the measured intensity, it can be seen that there are inconsistences in the case of the β-DIB reaction. We postulate that there are other species also present within the reaction mixture^93,94^. Through review of reaction databases, we note that there is likely 2-methylbutanal produced in the combustion process^32,95^. In the case of the C_4_H_6_O channel, both 2-butenal and dimethylketene are the two most likely isomers responsible for the observed signal from the β-DIB reaction. Of the C_4_H_8_O isomers, the ignition of β-DIB strongly favors the formation of 2-buten-1-ol over n-butanal by a 3.8:1 ratio; while the ignition of α-DIB is equally likely to produce n-butanal, tetrahydrofuran and 2-buten-1-ol. α-DIB ignition also exhibits less selectivity for the formation of any particular species within the C_3_H_6_O family of isomers as evidenced by near equal production of 2-propen-1-ol, acetone, and methyloxirane; while β-DIB oxidation tends to produce more acetone and methyloxirane.

#### Non-oxygenated intermediates

**Figure 6:**
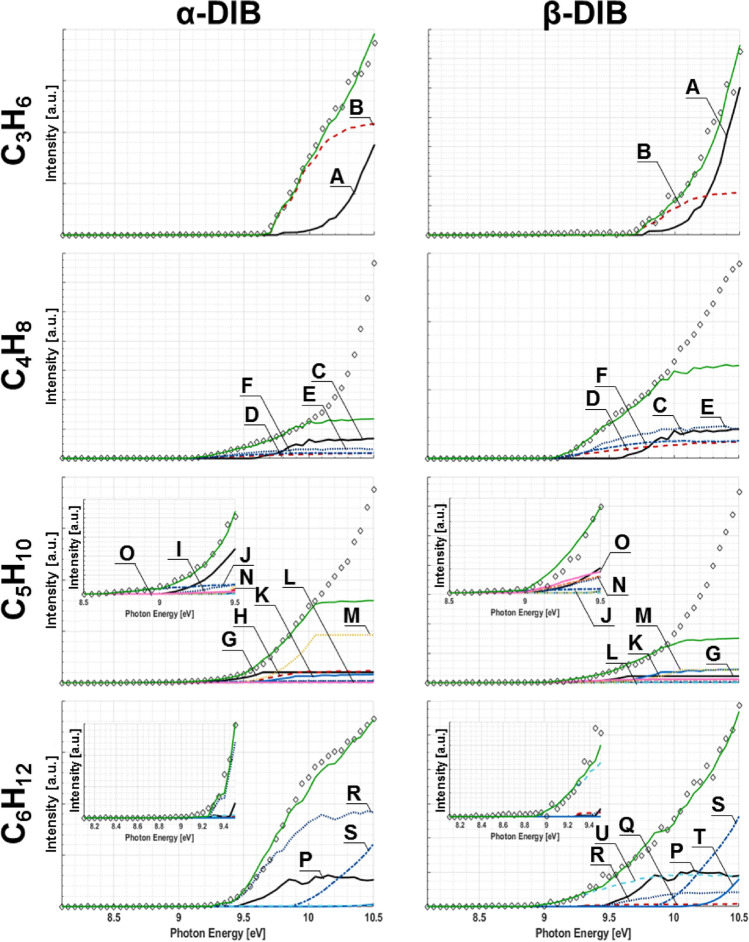
Intensity curves measured “◇” for non-oxygenated species in α- and β-DIB reactions. Colored traces correspond to those for species from Table 3, while green is reserved for the fit to experiment. Where appropriate, the inserts show magnified plots.

Several non-oxygenated intermediates have also been identified: C_6_H_12_, C_5_H_10_, C_4_H_8_, and C_3_H_6_. Intensity curves, *SS*_*i*_(E), and *SS*_*m/z*_*(E)* are presented in Fig. 6, with respective $SF$ and ΔH_f-(g)_ values for the corresponding species shown in Table 3. For the C_3_H_6_ channel, the α-DIB reaction forms propene and cyclopropane with a 1:1.4 ratio, whereas the β-DIB reaction results in a ratio of 1:6.2; indicating that there is a stronger reliance by the α-DIB reactions on allyl radical chemistry (•CH=CHCH_2_). For the C_4_H_8_ channel, the α-DIB reaction strongly favors the formation of 1-butene followed by approximately half as much isobutene and a quarter as much of the cis/trans-2-butenes, while the β-DIB reaction forms isobutene and 1-butene in equal amounts followed by half as much of each of the cis/trans-2-butenes. In the case of the C_6_H_12_ isomers, it can be seen that the α-DIB reaction favors formation of 3,3-dimethyl-1-butene by a factor of ~ 2.9 and ~ 2.6 over 1-hexene and cyclohexane, respectively; while the β-DIB reaction produces nearly equal concentrations of cyclohexane and methylcyclopentane, both of which are greater than 1-hexene or trans-2-hexene. Finally, the C_5_H_10_ channel shows similarities in its major isomers as both α-DIB and β-DIB reactions produce high concentrations of ethyl-cyclopropane and 1,1-dimethyl-cyclopropane, however, the ratios between these two products are 5:1 in the case of α-DIB reaction and only 2:1 in the case of β-DIB reaction.

### Insights into DIB reaction mechanisms

**Figure 7:**
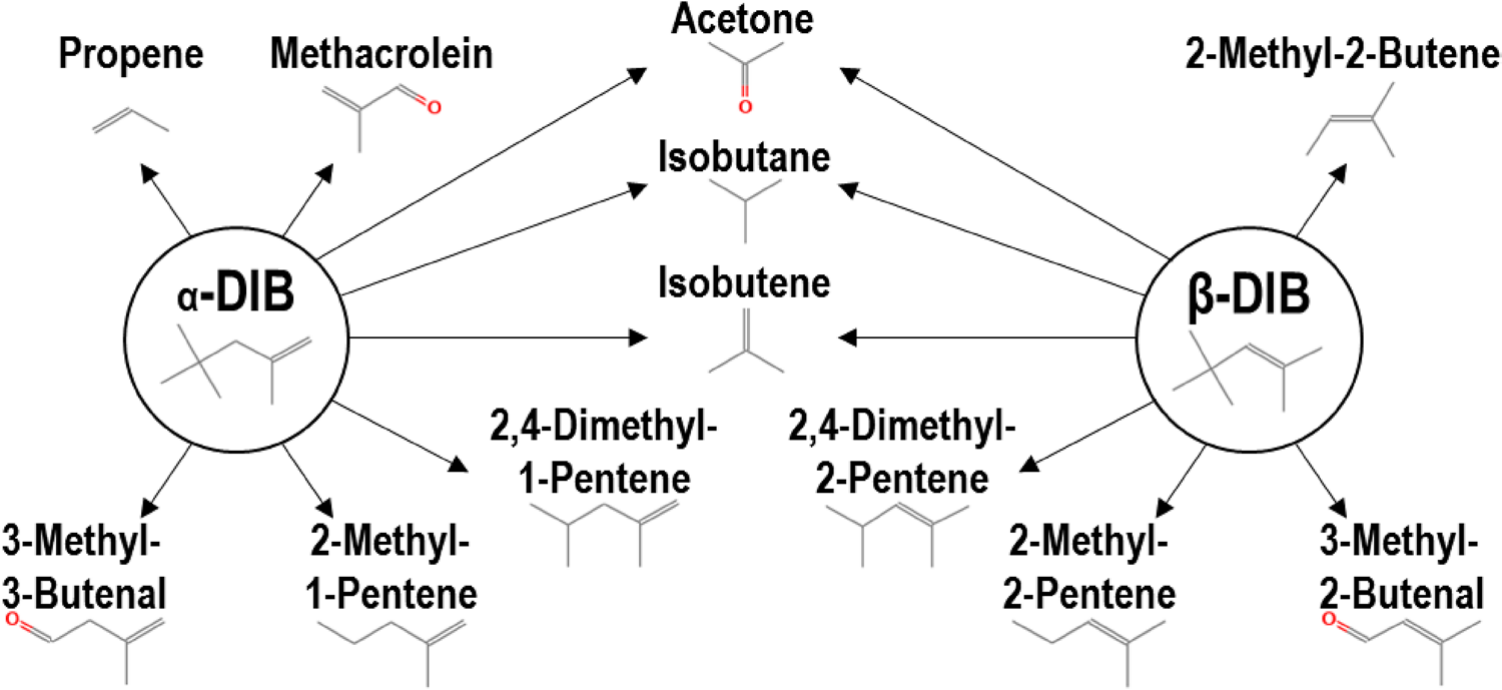
Identified byproducts from initiating reactions of α- and β-DIB.

The existing DIB reaction mechanism by Metcalfe et al.^32^ provides reasonable estimates for engineering applications, however, it may be improved upon to better represent the underlying chemical processes. Within said Metcalfe mechanism: (i) there is a predominance of successive series of N + M → N’ + M, N’ + M → N’’ + M style of reactions, (ii) the existing mechanism includes pathways that are energetically unlikely to occur as single step reactions, in which β-DIB undergoes an H abstraction to form an α-DIB derived radical, and (iii) while DIB may undergo dissociation reactions at elevated temperatures, DIB is, however, more likely to undergo bimolecular collision initiated reactions. In this study, we have identified several improvements to enhance the chemical description of the mechanism using both the data obtained via photoionization mass spectrometry and via evaluation of the available literature data. In Fig. 7, species that have been either identified directly from intensity plots or from the presence of a signal at a given m/z channel are depicted. Additionally, for ultra-fuel-rich flames, a soot sub-mechanism should be included^16^.

Both isomers easily enable the direct formation of acetone, isobutane and isobutene as the DIBs undergo oxidation. Even still, the position of the C=C bond will dictate the probability of the compounds that may form within the specific isomer’s reaction pathways. This is most clearly pronounced via the isomer pairs of 2,4-dimethyl-(1 or 2)-pentene, 2-methyl-(1 or 2)- pentene, and 3-methyl-(2 or 3)-butenal, which all retain the C=C bond as well as its adjacent associated structures in the parent compound. However, there are some subtle differences observable, wherein there is facile formation of propene and methacrolein in the α-DIB reactions, while 2-methyl-2-butene may be formed from the β-DIB reaction; such propene presence indicates a tendency by the α-DIB to more likely form polycyclic aromatic hydrocarbons (PAH)^96^. It is worth noting the difference in the Metcalfe et al. mechanism^32^, in which the 2,4-dimethylpentane radical as an initial product of DIB combustion is presumed to be of high importance given the fact that, comparatively, facile formation of 2,4-dimethyl-(1 or 2)-pentene is suggested in this work.

**Figure 8:**
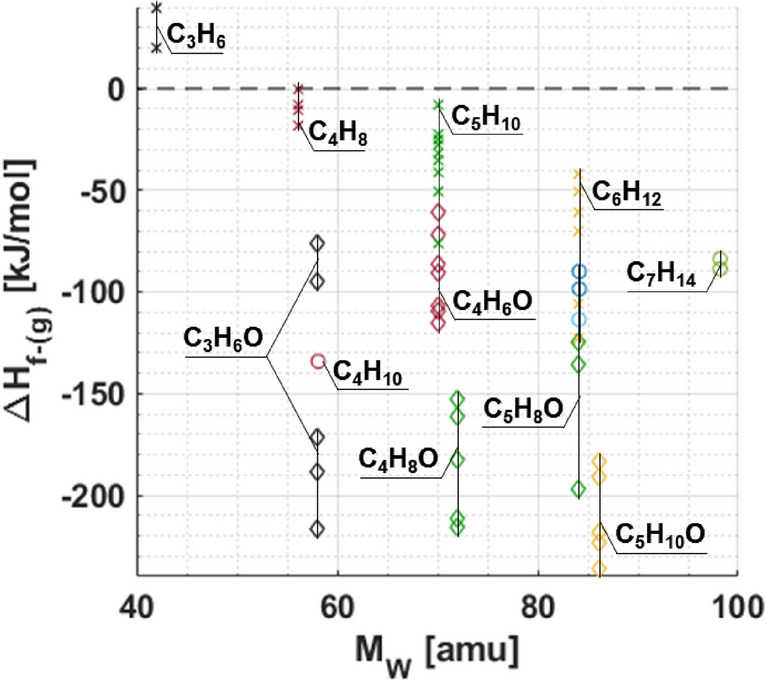
Standard enthalpy of formation versus molecular mass of various species and those of the intermediate species identified from DIB oxidation reactions. Colors shown are to differentiate compositions at near mass channels.

From the species identified in “Identified intermediates of DIB ignition” section, as well as others presented within Fig. 7, Fig. 8 shows standard enthalpy of formation in the gas phase (ΔH_f-(g)_) for the relevant intermediates; where the data points “X” correspond to non-oxygenated species, while those delineated by “◊” correspond to oxygenated species plotted against their corresponding molecular weight M_w_ [amu]. Species identified to form via abstraction processes are marked with an “o”. For reference, we note that α- and β-DIB have an estimated ΔH_f-(g)_ values of − 110.9 kJ/mol and − 112.4 kJ/mol, respectively^32^. Apart from the species that are isomers of C_3_H_6_, each of the identified compositional isomers have values of ΔH_f-(g)_ of < 0. At a given M_w_, oxygenated isomers are associated with dramatically lower ΔH_f-(g)_ values than their non-oxygenated counterparts^97^.

On comparing the ΔH_f-(g)_ values with the SF values in Tables 2 and 3, it can be seen that the yield of an non-oxygenated species is larger at a given mass channel for α-DIB, which is more resistant to ignition. Thus, indicating that oxygenation reactions are predominantly responsible for the heat release and that the resulting non-oxygenated fragments coincide with sustainment of the reaction. For example, in the α-DIB reaction, there is a dramatically larger concentration of ethyl-cyclopropane than the yield of the entire assorted C_4_H_6_O varieties; and similarly for 1-butene, compared to the C_3_H_6_O varieties. Conversely for the β-DIB reaction, there is a tendency to form C_3_H_6_O and C_4_H_8_O with relatively equal amounts of 2,3-dimethyl-2-butene and 3-methylbutanal, which are likely occurring as a result of initial reactions with the fuel.

#### α-DIB initial reactions

From the photoionization data (shown in higher detail in the Supporting Info), it can be asserted that α-DIB may undergo several single or multi-step reactions, in which a compound with mass greater than that of the parent fuel compound results. Elementary reactions are described in Table 4. Reactions R1 through R3 postulate the formation of C_7_H_13_O, C_7_H_14_O, or C_9_H_18_. Several other species may also be formed in significant quantities such as C_7_H_15_O, C_6_H_10_O_2_, and C_8_ oxygenates. In addition to R1-R3, there are also numerous reactions that lead to the formation of compounds with lower molecular weights. From Metcalfe et al*.*^32^, it is known that α-DIB may undergo a dissociation reaction to form the tert-butyl (C_4_H_9_) and isobutene (C_4_H_8_) radicals (R4), or the tert-butyl radical and stabilized isobutene as in R5.

**Table 4:**
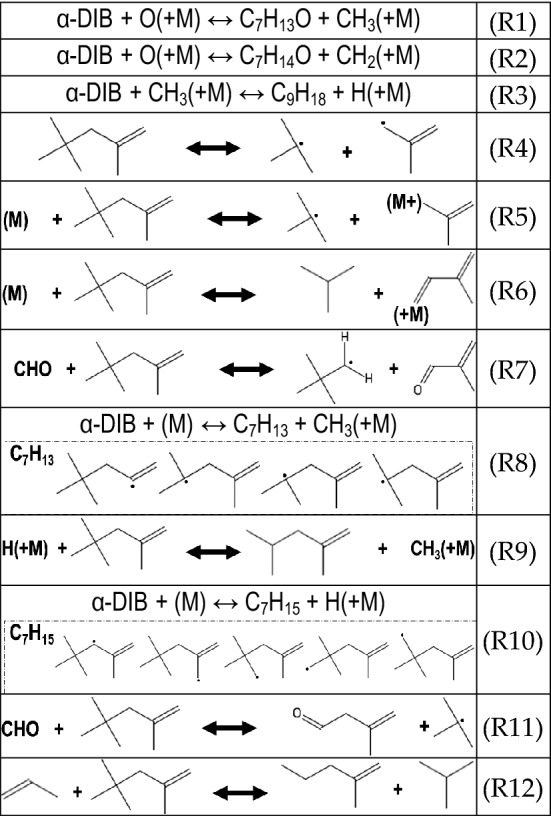
Reactions pertinent to α-DIB.

Given the relative relative signal attributable to mass channels corresponding to C_4_H_8_ and C_4_H_9_, it may be assumed that under our test conditions, R4 and R5 are most sensitive to α-DIB oxidation reactions in determining the overall fate of the system. Such a statement may also be validated by recognizing the fact that the indicated common bond that is broken within the parent species has the lowest observed bond dissociation energy (BDE). Of additional importance to the C_4_ chemistry is the magnitude of the C_4_H_10_ signal, which is likely a result of the isobutane that forms from R6, among other reactions, and possibly other butanes. Alternatively, through R7, a carbonyl olefin metathesis reaction enables the formation of a 2,2-dimethylbutane radical. Through R8, the α-DIB may undergo methyl abstraction forming one of four distinct C_7_H_13_ radicals and a methylated byproduct. If an H containing species can interact with α-DIB, then a 2,4-dimethyl-1-pentene could subsequently be formed in R9, and an important secondary pathway for such a species would be hydrogenation of the C_7_H_13_ species. R10 depicts H cleavage from a C-H bond in α-DIB forming one of 5 possible radical conformers, in which the olefinic bond is preserved. The formation of 3-methyl-3-butenal and 2-methyl-1-pentene are enabled through methyl abstractions^29^, and combination of the fuel compound with either an aldehyde or propene, through R11 and R12.

#### β-DIB initial reactions

β-DIB’s vinylic bond enables subsequent reactions to form a wider array of species than in α-DIB as the outer electronic orbitals of the former molecule have a greater reaction cross section thus enabling its lower auto ignition temperature^98^. β-DIB is more likely to form species with greater masses, several of which can be oxygenated via R13 through R17, as shown in Table 5. Through R18, the formation of 5 potential radicals, which produce a complementary methylated species is possible via the abstraction of the corresponding methyl group in the β-DIB. From comparisons of the BDEs, the most likely methyl group to be abstracted corresponds to that from the tertiary substituted carbon (see Fig. 1)^29^. β-DIB reaction is also rich in C_4_ chemistry. As seen in the scission reactions R19 and R20, formation of either isobutene and tert-butyl radical, or isobutane and isobutene radical is possible. In addition, 2,4-dimethyl-2-pentene could also be formed via R21. Lastly, β-DIB may readily lose an H from one of the five methyl substituents (R22). Through combination with a methyl radical, β-DIB may form 2-methyl-2-butene (R23). Consideration of BDEs indicates that the most likely reactions for the formation of 2-methyl-2-pentene and the direct formation of 3-methyl-2-butenal is via R24 and R25, respectively^29^.

**Table 5:**
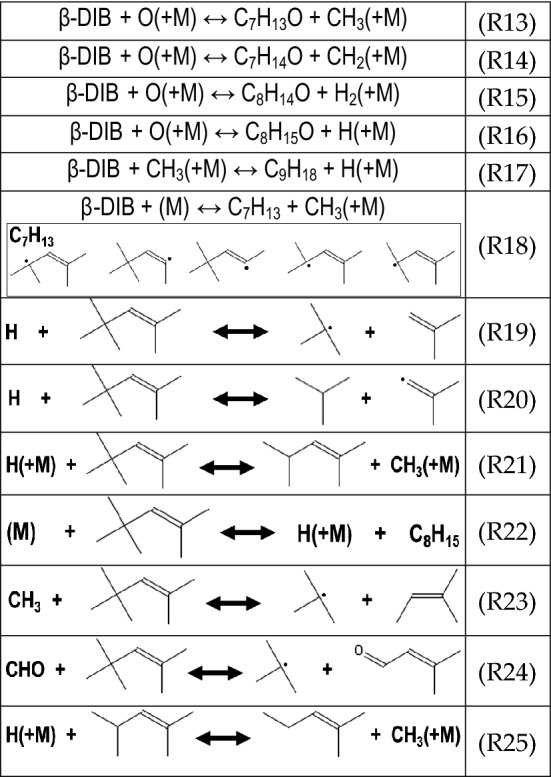
Reactions pertinent to β-DIB.

#### Other reactions key to DIB combustion

From the initial oxidation patterns of both the parent compound isomers, one can deduce some striking similarities in the reaction processes. These reactions are shown in Table 6. Following initial branching reactions, both fuels produce an array of butenes, which can subsequently form propene and other low-molecular weight intermediates, prior to the final CO_2_ and H_2_O equilibrium^99,100^. As there appears to be an extensive reliance on the tert-butyl radical chemistry in DIB combustion, it would be appropriate to include this chemistry to other existing mechanisms, such as that of Yasunaga et al.^101^, and De Bruycker et al.^102^. Of particular importance for DIB combustion is the ability of isobutylene to interact with methyl or methylene radicals as shown in R26 and R27.

**Table 6:**
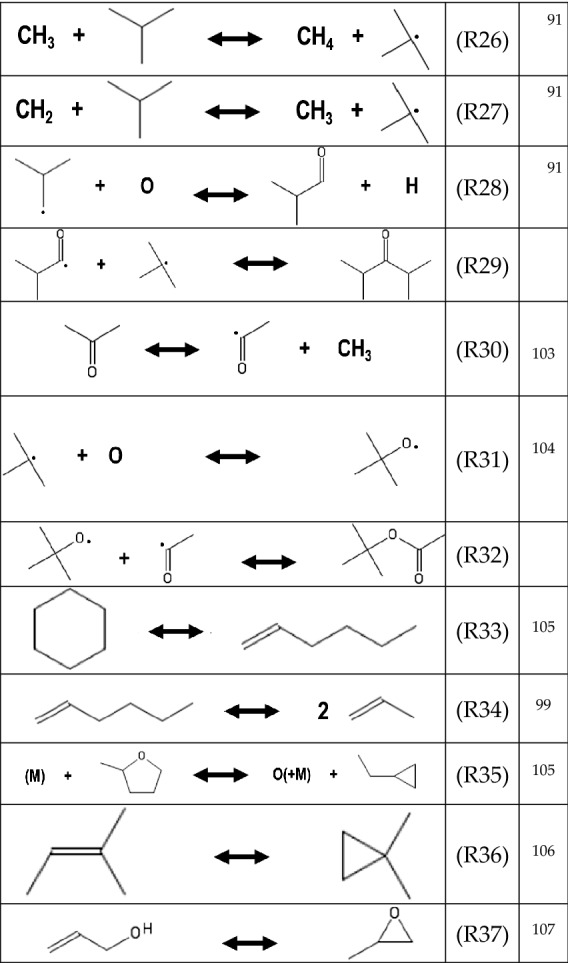
Reactions identified that are pertinent to both α- and β-DIB.

The high abundance of C_7_H_14_O indicates that there may be compounds formed via processes other than those specified in R2. For example, in R28 it is possible that radicals of isobutylene and O may react to form 2-methylpropanal. Similarly, it is possible that isobutyraldehyde and the tert-butyl radical can combine to form 2,4-dimethyl-3-pentanone, C_7_H_14_O via R29. Another species of note for both DIB isomer reactions is C_6_H_10_O_2_. Though an analysis of its intensity curve was not possible due to the absence of its literature PICS, it is a reasonable expectation that isopropyl acrylate forms via R30 and R31.

Cyclic species may also be abundant within the reaction environment in the form of three, five, and six-membered ring compounds, which may include heterocycles. Thus, in addition to the inclusion of C_4_ chemistry, it would appear that consideration of cyclic species be given in the reaction mechanism for DIB^108^. We identified the possibility of cyclohexane formation from propene via R33 and R34^99,105^. In this work, 2-methyltetrahydrofuran was identified, which we assume is formed from either 2,4-dimethyl-(1 or 2)-pentene, 2,4-dimethyl-3-pentanone, or isopropyl acrylate. Via R35, methyltetrahydrofuran may lead to the formation of cyclopropane containing species^105^. Cyclopropanes are known to dissociate as shown in R36 and R37 via ring opening reactions.

## Conclusions

Diisobutylene (DIB) is a high-performance biofuel compound selected by the U.S. Department of Energy’s Co-Optima project and can be readily produced from bio-derived sugars. DIB commonly exists as one of two isomers: 2,4,4-trimethyl-1-pentene (α-DIB) or 2,4,4-trimethyl-2-pentene (β-DIB) and is of interest as a drop-in additive for gasoline fuels. Within this work, the ignition behavior of stoichiometric composition, using either the α- or β-diisobutylene (DIB) isomer, has been studied using a jet stirred reactor (JSR) in conjunction with photoionization mass spectrometry setup available at the ALS. Isomeric detection of various intermediates formed during oxidation of these compounds was achieved. Using the two most prominent isotopomers of α- & β-DIB of C_8_H_16_ and C_7_^13^CH_16_, relative intensity curves for each at the primary mass channel of m/z = 112.2 were obtained. The nature of the olefinic carbon (allylic versus vinylic) dramatically affects the distribution of molecular fragments observed during ignition. These distributions can be directly linked to the observed combustion characteristics of each compound. This work suggests that the DIB reaction mechanism should include detailed sub-mechanisms for the tert-butyl species as well as for heterocyclic three, five, and six-atom rings. A better understanding of the differences in ignition properties from a molecular level will facilitate the development of engines specifically optimized for these types of fuels and will facilitate the reduction of carbon emissions in the future.

## Supplementary information

Supplementary Information.
