# Supplementary material for: Elucidating the differences in oxidation of high-performance α- and β- diisobutylene biofuels via Synchrotron photoionization mass spectrometry
Source: Sci Rep. 2020 Dec 11;10:21776. doi: 10.1038/s41598-020-76462-y (PMC7733457; doi:10.1038/s41598-020-76462-y)
Supplement: Supplementary file 1 — Supplementary Information. [file 41598_2020_76462_MOESM1_ESM.docx]

# Supplementary File

**Elucidating the differences in oxidation of high-performance α- and β- diisobutylene biofuels via synchrotron photoionization mass spectrometry**

**Anthony Carmine Terracciano^1,2^, Sneha Neupane^1,2, #^, Denisia Popolan-Vaida^3^, Richard G. Blair^2,4^, Nils Hansen^5^, Ghanshyam L. Vaghjiani^6^, Subith S. Vasu^1,2, *^**

^1^Mechanical and Aerospace Engineering Department, University of Central Florida, 4000 Central Florida Blvd., Orlando, FL 32816-2450, USA

^2^Center for Advanced Turbomachinery and Energy Research (CATER), University of Central Florida, 4000 Central Florida Blvd., Orlando, FL 32816-2450, USA

^3^Department of Chemistry, University of Central Florida, 4000 Central Florida Blvd., Orlando, FL 32816-2450, USA

^4^Florida Space Institute, University of Central Florida, 4000 Central Florida Blvd., Orlando, FL 32816-2450, USA

^5^Combustion Research Facility, Sandia National Laboratories, P.O. Box 969, MS 9055, Livermore, CA 94551, USA

^6^In-Space Propulsion Branch, Rocket Propulsion Division, Aerospace Systems Directorate, Air Force Research Laboratory, AFRL/RQRS, Edwards AFB, CA 93524

**^#^**Presently a research scientist at Oak Ridge National Lab, TN, USA

**Keywords:** VUV Multiplexed Mass Spectrometry; Co-Optima Biofuels; Ignition Reaction Kinetics; Photoionization; 2,4,4-Trimethyl-1-Pentene; 2,4,4-Trimethyl-2-Pentene; Jet Stirred Reactor

**^*^**Corresponding author: [subith@ucf.edu](mailto:subith@ucf.edu)

# Appendix 1

Photoionization data for the DIB isomers.

|  |  | α-DIB | | |  | β-DIB | | |
| --- | --- | --- | --- | --- | --- | --- | --- | --- |
|  |  | **Intensity** | |  |  | **Intensity** | |  |
| eV |  | **C_8_** | **C_7_^13^C** | **C_8_: C_7_^13^C** |  | **C_8_** | **C_7_^13^C** | **C_8_: C_7_^13^C** |
| 8.10 |  | 0 | 0 | 0.00 |  | 0 | 0.0 | 0.00 |
| 8.15 |  | 0.0 | 0.0 | 0.00 |  | 0.0 | 0.0 | 0.00 |
| 8.20 |  | 0.0 | 1.0 | 0.00 |  | 0.0 | 0.0 | 0.00 |
| 8.25 |  | 0.0 | 2.0 | 0.00 |  | 0.0 | 0.0 | 0.04 |
| 8.30 |  | 0.0 | 3.0 | 1.32 |  | 0.0 | 0.0 | 0.08 |
| 8.35 |  | 0.0 | 4.0 | 0.00 |  | 0.0 | 0.0 | 0.09 |
| 8.40 |  | 0.0 | 5.0 | 0.00 |  | 0.1 | 0.0 | 0.09 |
| 8.45 |  | 0.0 | 6.0 | 0.43 |  | 0.3 | 0.0 | 0.09 |
| 8.50 |  | 0.0 | 7.0 | 0.08 |  | 0.8 | 0.1 | 0.08 |
| 8.55 |  | 0.0 | 8.0 | 0.10 |  | 1.5 | 0.1 | 0.08 |
| 8.60 |  | 0.0 | 9.0 | 0.06 |  | 2.2 | 0.2 | 0.08 |
| 8.65 |  | 0.0 | 10.0 | 0.07 |  | 3.0 | 0.3 | 0.09 |
| 8.70 |  | 0.0 | 11.0 | 0.08 |  | 4.2 | 0.3 | 0.08 |
| 8.75 |  | 0.1 | 12.0 | 0.08 |  | 5.3 | 0.4 | 0.09 |
| 8.80 |  | 0.2 | 13.0 | 0.08 |  | 6.2 | 0.5 | 0.08 |
| 8.85 |  | 0.8 | 14.0 | 0.08 |  | 7.4 | 0.6 | 0.08 |
| 8.90 |  | 2.1 | 15.0 | 0.08 |  | 8.7 | 0.7 | 0.08 |
| 8.95 |  | 3.6 | 16.0 | 0.08 |  | 10.4 | 0.8 | 0.08 |
| 9.00 |  | 5.3 | 17.0 | 0.08 |  | 11.7 | 1.0 | 0.08 |
| 9.05 |  | 7.4 | 18.0 | 0.08 |  | 13.3 | 1.1 | 0.08 |
| 9.10 |  | 9.8 | 19.0 | 0.08 |  | 14.9 | 1.2 | 0.08 |
| 9.15 |  | 11.8 | 20.0 | 0.08 |  | 16.5 | 1.3 | 0.08 |
| 9.20 |  | 13.8 | 21.0 | 0.08 |  | 18.3 | 1.5 | 0.08 |
| 9.25 |  | 16.2 | 22.0 | 0.08 |  | 19.7 | 1.6 | 0.08 |
| 9.30 |  | 18.3 | 23.0 | 0.08 |  | 21.2 | 1.7 | 0.08 |
| 9.35 |  | 20.6 | 24.0 | 0.08 |  | 22.8 | 1.8 | 0.08 |
| 9.40 |  | 22.9 | 25.0 | 0.08 |  | 24.5 | 1.9 | 0.08 |
| 9.45 |  | 25.9 | 26.0 | 0.08 |  | 25.7 | 2.1 | 0.08 |
| 9.50 |  | 28.6 | 27.0 | 0.08 |  | 26.5 | 2.1 | 0.08 |
| 9.55 |  | 32.0 | 28.0 | 0.08 |  | 27.7 | 2.2 | 0.08 |
| 9.60 |  | 35.0 | 29.0 | 0.08 |  | 28.6 | 2.3 | 0.08 |
| 9.65 |  | 37.4 | 30.0 | 0.08 |  | 29.5 | 2.4 | 0.08 |
| 9.70 |  | 40.4 | 31.0 | 0.08 |  | 30.4 | 2.5 | 0.08 |
| 9.75 |  | 43.5 | 32.0 | 0.08 |  | 33.1 | 2.6 | 0.08 |
| 9.80 |  | 46.0 | 33.0 | 0.08 |  | 34.8 | 2.8 | 0.08 |
| 9.85 |  | 48.3 | 34.0 | 0.08 |  | 36.0 | 2.8 | 0.08 |
| 9.90 |  | 50.9 | 35.0 | 0.08 |  | 38.3 | 3.1 | 0.08 |
| 9.95 |  | 53.7 | 36.0 | 0.08 |  | 42.6 | 3.4 | 0.08 |
| 10.00 |  | 56.9 | 37.0 | 0.08 |  | 47.9 | 3.8 | 0.08 |
| 10.05 |  | 59.1 | 38.0 | 0.08 |  | 52.3 | 4.2 | 0.08 |
| 10.10 |  | 61.4 | 39.0 | 0.08 |  | 56.2 | 4.4 | 0.08 |
| 10.15 |  | 64.3 | 40.0 | 0.08 |  | 58.8 | 4.7 | 0.08 |
| 10.20 |  | 66.6 | 41.0 | 0.08 |  | 62.6 | 5.0 | 0.08 |
| 10.25 |  | 68.2 | 42.0 | 0.08 |  | 67.0 | 5.3 | 0.08 |
| 10.30 |  | 70.4 | 43.0 | 0.08 |  | 69.1 | 5.5 | 0.08 |
| 10.35 |  | 73.9 | 44.0 | 0.08 |  | 70.8 | 5.7 | 0.08 |
| 10.40 |  | 77.5 | 45.0 | 0.08 |  | 72.9 | 5.8 | 0.08 |
| 10.45 |  | 81.6 | 46.0 | 0.08 |  | 73.0 | 5.8 | 0.08 |
| 10.50 |  | 84.6 | 47.0 | 0.08 |  | 72.9 | 5.9 | 0.08 |
| 10.55 |  | 87.5 | 48.0 | 0.08 |  | 76.9 | 6.2 | 0.08 |
| 10.60 |  | 88.9 | 49.0 | 0.08 |  | 81.5 | 6.6 | 0.08 |
| 10.65 |  | 92.3 | 50.0 | 0.08 |  | 86.1 | 7.0 | 0.08 |
| 10.70 |  | 94.6 | 51.0 | 0.08 |  | 88.4 | 7.2 | 0.08 |
| 10.75 |  | 96.7 | 52.0 | 0.08 |  | 89.7 | 7.2 | 0.08 |
| 10.80 |  | 97.6 | 53.0 | 0.08 |  | 91.3 | 7.4 | 0.08 |
| 10.85 |  | 97.2 | 54.0 | 0.08 |  | 91.8 | 7.4 | 0.08 |
| 10.90 |  | 100.0 | 55.0 | 0.08 |  | 93.4 | 7.6 | 0.08 |
| 10.95 |  | 98.7 | 56.0 | 0.08 |  | 98.1 | 7.7 | 0.08 |
| 11.00 |  | 98.6 | 57.0 | 0.08 |  | 100.0 | 7.8 | 0.08 |

# Appendix 2

Count normalized mass spectra for α- (A and C), and β-diisobutylene (B and D: at 10.5 eV ionization energy.

**
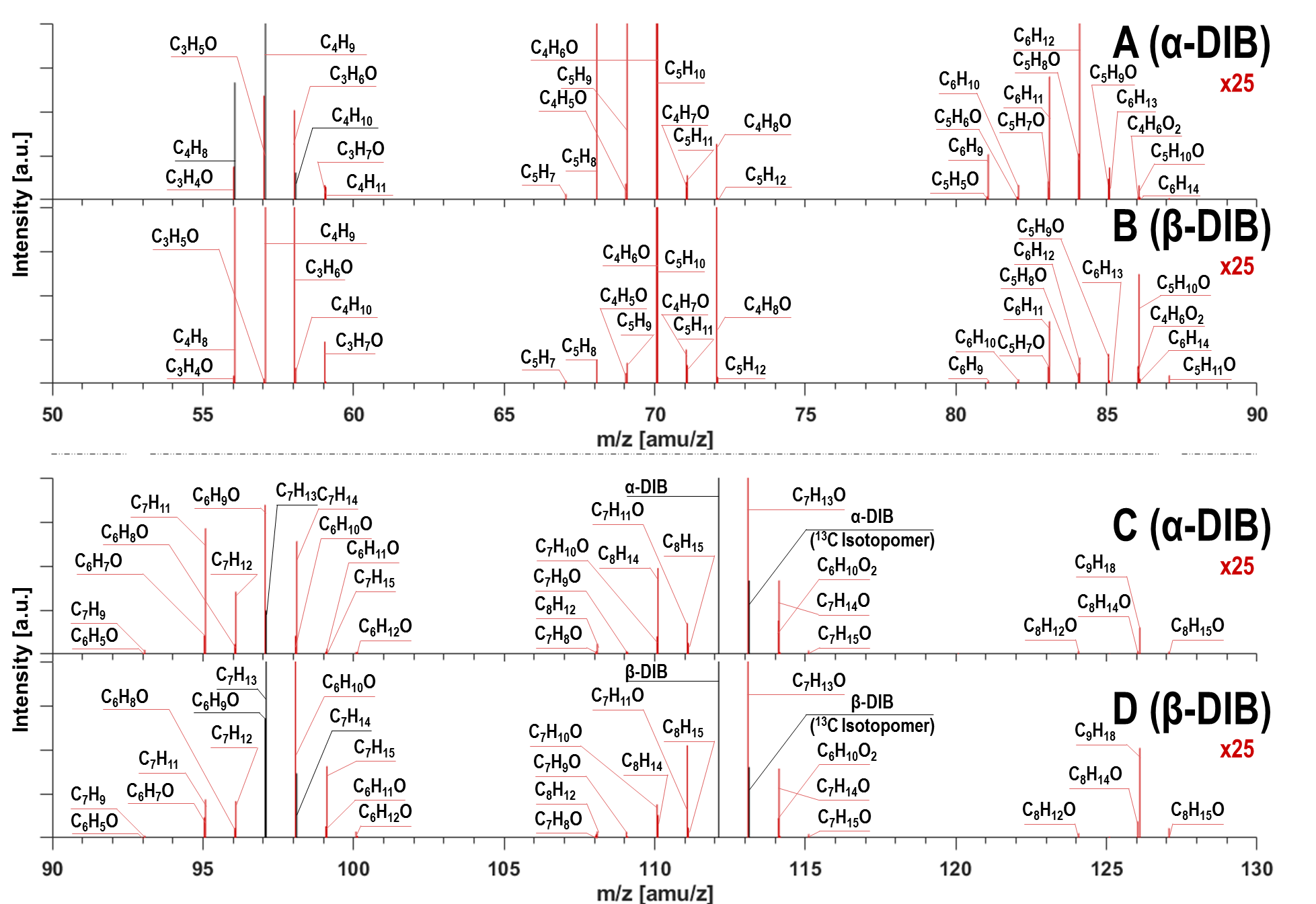
**
